# Transforming Stroke Diagnosis with Artificial Intelligence: A Scoping Review of Brainomix e-Stroke, Aidoc, RapidAI, and Viz.ai

**DOI:** 10.3390/medicina62030582

**Published:** 2026-03-19

**Authors:** Mateusz Dorochowicz, Arkadiusz Kacała, Aleksandra Tołkacz, Aleksandra Kosikowska, Maja Gewald, Maciej Guziński

**Affiliations:** 1 Department of General, Interventional and Neuroradiology, Wroclaw University Hospital, 50-367 Wrocław, Poland; 2 Department of Radiology, Wroclaw Medical University, 50-367 Wroclaw, Poland; 3 Department of General, Interventional and Neuroradiology, Wroclaw Medical University, 50-367 Wrocław, Poland; 4 Faculty of Medicine, Wroclaw Medical University, 50-367 Wrocław, Poland

**Keywords:** acute ischemic stroke, artificial intelligence, deep learning, automated detection, machine learning, stroke care, large vessel occlusion

## Abstract

*Background and Objectives*: Rapid diagnosis is fundamental to acute ischemic stroke management; however, access to neuroradiological expertise remains limited. This scoping review maps the diagnostic accuracy, workflow impact, and cost-effectiveness of leading AI platforms (Brainomix, Aidoc, RapidAI, and Viz.ai), characterizing industry and peer-reviewed metrics. *Materials and Methods*: Following PRISMA-ScR guidelines, we searched PubMed, Cochrane Library, and HTA repositories for studies (2019–2025). Using a PICO-based framework, 29 studies were included for thematic mapping of the technological landscape. *Results*: Twenty-nine studies were included. Platforms show high proximal LVO sensitivity (78–97%), while performance for distal/MVO and posterior circulation occlusions was more variable. RapidAI is frequently mapped using historical perfusion trial parameters; however, volumetric discrepancies with platforms like Viz.ai indicate outputs are not interchangeable. Brainomix shows extensive validation for automated NCCT ASPECTS in triage. Aidoc demonstrates operational advantages via worklist prioritization, while. Viz.ai is associated with door-to-puncture time reductions (11–25 min). Economically, cost-effectiveness is driven by improved functional outcomes and expanded access to thrombectomy, rather than labor substitution. *Conclusions*: AI platforms function as diagnostic safety nets and workflow optimizers. Reported roles, such as perfusion-centric analysis (RapidAI) or workflow coordination (Viz.ai), reflect current research trends rather than definitive technological superiority. Institutional selection should consider these evidence clusters alongside local validation and specific clinical priorities.

## 1. Introduction

Ischemic stroke is a leading cause of long-term disability and mortality worldwide, straining modern healthcare systems. The “Time is Brain” principle underscores that even slight delays in diagnosing ischemia can result in substantial loss of neural tissue. The effectiveness of reperfusion procedures, such as intravenous thrombolysis (IVT) or mechanical thrombectomy (MT), largely depends on the speed of diagnosis, which makes every minute critical [1,2]. Neuroimaging sits at the heart of the stroke pathway. Non-contrast computed tomography (NCCT) has become the diagnostic standard, often complemented by computed tomography angiography (CTA) and, in selected time windows, computed tomography perfusion (CTP) [2].

In clinical practice, especially during on-call shifts, the availability of experienced neuroradiologists to interpret these studies can be limited. Delays in image interpretation, the need for manual calculation of the ASPECTS score, or manual evaluation of vascular patency may prolong critical time metrics, such as door-to-needle time (DNT) and door-to-puncture time (DPT) [3,4].

The urgent need for more efficient triage in acute ischemic stroke has accelerated the clinical adoption of Clinical Decision Support Systems (CDSS) powered by AI and machine learning [5,6]. Current industry-standard platforms (notably Brainomix e-Stroke, Aidoc, RapidAI, and Viz.ai) now enable real-time CT analysis, which has been associated with reductions in selected workflow intervals. These systems equip stroke teams with critical automated insights, including the identification of large vessel occlusion (LVO) [7,8,9] and quantitative ASPECTS scoring (e.g., via e-ASPECTS) [10,11,12]. Furthermore, automated perfusion analysis now allows for rapid estimation of infarct core volume and penumbral tissue, which is vital for guiding treatment selection [13,14].

While AI systems have been rapidly integrated into stroke protocols, their overall clinical utility remains a subject of ongoing debate. To date, the evidence base is dominated by validation studies, a significant proportion of which are industry-sponsored or conducted by authors affiliated with software vendors, raising concerns regarding potential bias and limited generalizability [15,16,17,18]. At the same time, several performance claims stem from non-peer-reviewed sources, such as manufacturer press releases and marketing materials, which often report favorable performance of proprietary algorithms. This reliance on self-reported data can obscure the true clinical value of these tools, emphasizing the need for independent, methodologically rigorous comparative research. Head-to-head comparisons within identical patient cohorts are still limited [19,20].

Despite advancements, diagnostic accuracy remains variable, especially for medium vessel occlusions (MVO) and reliable perfusion mapping, as reported in peer-reviewed studies [21] and noted in vendor-related reports [22,23]. Significant discrepancies in reported diagnostic accuracy continue to exist, particularly in perfusion analysis, which is a key determinant of patients’ eligibility for reperfusion therapy [13,14,24,25].

This scoping review aims to map the available evidence regarding the clinical utility of AI-based tools in acute stroke imaging. Beyond identifying reported diagnostic accuracy for large and medium vessel occlusions (LVO/MVO) [19,26], we characterize the landscape of workflow metrics [3] and economic evaluations derived from recent HTA reports and NICE guidelines [27,28,29]. Crucially, the functional roles often attributed to specific platforms in this narrative are descriptive of the current evidence base and existing research trends; they are not intended to imply the definitive superiority of any single platform. Rather than generating pooled quantitative estimates, the objective is to summarize methodological patterns and evidence gaps to provide a transparent framework for clinical decision-making.

## 2. Materials and Methods

This scoping review was conducted and reported in accordance with the PRISMA-ScR (Preferred Reporting Items for Systematic Reviews and Meta-Analyses Extension for Scoping Reviews) statement [30] and methodological guidance from the Joanna Briggs Institute [31]. We carefully selected studies based on predefined inclusion and exclusion criteria, structured according to the PICO framework. The target population included patients with suspected acute ischemic stroke undergoing CT-based neuroimaging (including non-contrast CT, CT angiography [CTA], and/or CT perfusion [CTP]). Our primary intervention focused on the clinical application of commercially available AI platforms (Brainomix e-Stroke https://www.brainomix.com, Aidoc https://www.aidoc.com, RapidAI https://www.rapidai.com, Viz.ai https://www.viz.ai) for automated image analysis. These systems were compared against standard radiological assessment (as the reference standard), other AI software in head-to-head studies, or pre-implementation historical data. We categorized outcomes of interest into four key domains: (1) diagnostic accuracy for vessel occlusions; (2) workflow metrics, notably door-to-needle (DNT) and door-to-puncture (DPT) times; (3) imaging parameters, including ASPECTS scoring agreement and ischemic core volume; and (4) economic outcomes, including cost-effectiveness of AI implementation.

A systematic literature search was conducted across PubMed/MEDLINE, PubMed Central (PMC), and the Cochrane Library. In addition, ResearchGate was used as a supplementary source to identify grey literature and author-shared manuscripts not yet indexed in major repositories, ensuring a comprehensive evidence base. Grey literature was included only when it reported sufficient methodological detail to permit independent critical appraisal (e.g., cohort size, reference standard, predefined endpoints, and basic statistical analysis). These sources were used primarily to characterize emerging technological developments, whereas clinical interpretations and conclusions were anchored in peer-reviewed and independent evidence. Further evidence was gathered through a targeted search of Health Technology Assessment (HTA) agencies, notably from NICE, and selected conference proceedings. The search was restricted to studies published between 2019 and 2025 to reflect contemporary AI algorithms and clinical imaging workflows. Our strategy was based on a combination of appropriate keywords and MeSH terms, applied using Boolean operators to optimize the retrieval of relevant studies, and covering three main areas: AI technologies and names of AI systems (“Artificial Intelligence”, “Deep Learning”, “Automated Detection”, “RapidAI”, “Viz.ai”, “Brainomix e-Stroke”, “Aidoc”, “e-ASPECTS”); stroke diagnostics and pathology (“Acute Ischemic Stroke”, “Large Vessel Occlusion” [LVO], “Medium Vessel Occlusion” [MVO], “CT Perfusion” [CTP], “CT Angiography” [CTA]); and clinical and operational outcomes (“Door-to-puncture time”, “Workflow efficiency”, “Diagnostic accuracy”, “Cost-effectiveness”). The review was restricted to articles published in English.

Eligible study designs comprised retrospective and observational studies, external validation studies, and economic evaluations, including HTA reports. We excluded single case reports, animal studies, and materials of a purely promotional nature or lacking verifiable methodological detail. Conference abstracts were generally excluded due to limited methodological transparency; however, an exception was made for selected Late Breaking Abstracts. These were included only if they provided timely, essential evidence and reported essential methodological elements, including cohort size, reference standard, predefined endpoints, and basic statistical analysis. Evidence derived from conference materials was considered to be of lower certainty and interpreted accordingly.

**Figure 1:**
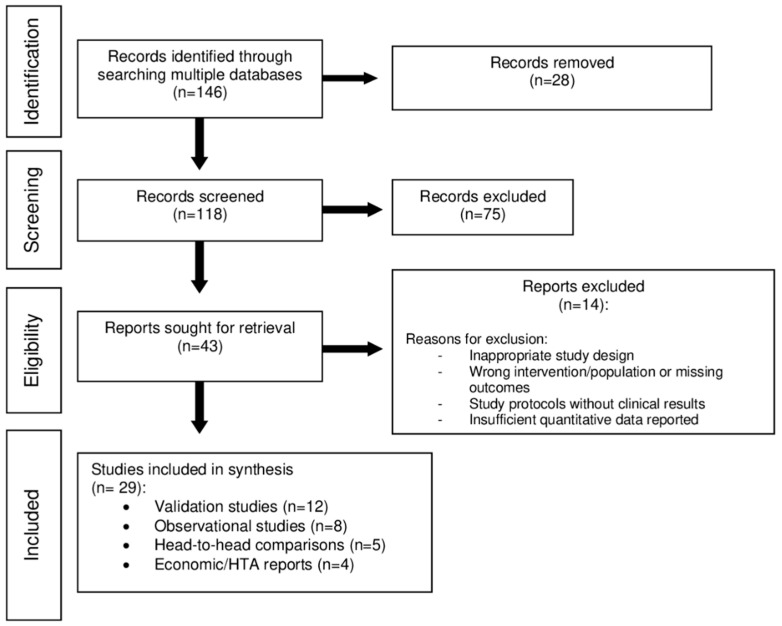
PRISMA Flow Diagram for Scoping Reviews.

Study selection was performed in two stages. To ensure methodological rigor, title and abstract screening, followed by full-text assessment, was performed independently by three reviewers. Any discrepancies regarding study inclusion were resolved through consensus or, where necessary, through consultation with a senior reviewer. The complete selection workflow is illustrated in the PRISMA Flow Diagram for Scoping Reviews (Figure 1). Data extraction was conducted using a standardized framework and included study characteristics, patient populations, AI platform and software version, comparator methodology, reported outcomes, and funding sources. We collected information on funding and industry involvement to account for potential conflicts of interest.

Given the substantial heterogeneity of included study designs (retrospective validations, real-world implementation studies, economic models, and HTA reports), variability in endpoints, and rapid technological evolution of AI platforms, a scoping review methodology was considered most appropriate. The aim was to map the breadth and characteristics of the available evidence, and identify methodological gaps, rather than to conduct quantitative pooling or formal comparative effectiveness analysis. Findings were organized thematically to reflect how these tools are currently represented in the literature, focusing on: (1) the accuracy of detecting vascular occlusions; (2) the reliability of automated perfusion and ASPECTS scoring; and (3) associations with workflow-related time metrics relevant to reperfusion therapy.

A formal risk-of-bias assessment (e.g., ROBIS or QUADAS-2) was not performed, as the objective was evidence mapping, rather than graded effectiveness synthesis. The protocol was not prospectively registered.

## 3. Results

### 3.1. Characteristics of the Included Studies

The 29 included studies demonstrated substantial methodological heterogeneity. Retrospective designs predominated. In most studies, archived CT/CTA examinations were processed with AI algorithms and benchmarked against independent reference standards (e.g., DSA or expert radiological consensus). Consequently, these designs allow for a direct assessment of diagnostic performance metrics (sensitivity and specificity). However, they reflect the realities of acute on-call practice only to a limited extent. In more recent publications, there is a growing proportion of real-world evidence studies, focusing not only on detection accuracy but also on the impact of AI implementation on clinical workflow and patient triage within stroke networks, including multicenter models (e.g., the NHS). Over time, an evolution of endpoints is also apparent: from a primary focus on diagnostic accuracy in 2019–2022, through greater emphasis on time-based and organizational metrics in 2023–2024, to increasing interest in more challenging clinical problems in 2024–2025, such as the detectability of medium/distal vessel occlusions (MVO) and the comparability of perfusion outputs across vendors. In the current literature landscape, most studies focused on Viz.ai and RapidAI software, reflecting their widespread clinical adoption in the U.S. market and their early inclusion in major clinical trials. Studies evaluating Brainomix e-Stroke originated mainly from European centers (the United Kingdom, NHS networks), whereas Aidoc was analyzed primarily in the context of broader radiology applications. These regional and functional research clusters significantly influence the reported utility of each platform. Of the 29 included studies, 4 (13.8%) were identified as having direct industry sponsorship or involving authors with formal affiliations to the software vendors. The remaining 25 (86.2%) studies were categorized as independent, including investigator-initiated trials, academic retrospective validations, and official Health Technology Assessment (HTA) reports.

### 3.2. Diagnostic Accuracy in Detecting Vascular Occlusions (LVO and MVO)

Occlusion detection is the most extensively discussed topic in the current literature, but the evidence is highly heterogeneous. This variance is largely driven by thrombus location; diagnostic outcomes differ substantially between proximal M1 segments and more distal M2/M3 branches, with further performance shifts observed between the anterior and posterior circulations. In the context of large vessel occlusions (LVO; ICA/M1), current algorithms demonstrate high diagnostic reliability in identifying proximal lesions. The reported high diagnostic accuracy (sensitivities typically ranging from 78% to 97% and specificities from 74% to 97%) is derived from a mix of independent validations and vendor-initiated cohort studies [7,8,9]. A direct comparison of Aidoc and RapidAI provides further support for these findings. In a retrospective analysis involving 50 patients, no statistically significant differences were found in diagnostic accuracy for LVO detection between these tools (true positive rates of 78% and 74%, respectively). However, the study highlighted limitations in sensitivity, with both platforms exhibiting considerable false negative rates (22–26%), suggesting that physician oversight remains essential regardless of the AI tool used [19]. Similar evidence supports the use of Brainomix; validation studies of its e-CTA module show high diagnostic accuracy for automated LVO detection, performing on par with competing platforms [32]. Furthermore, real-world evidence from the UK NHS supports the clinical utility of this solution, highlighting its role in streamlining patient triage for thrombectomy, particularly when integrated with e-ASPECTS assessment [24,33]. 

Despite these strong performance metrics, independent analyses across major systems evaluated by NICE (RapidAI, Viz.ai, Brainomix) report a non-negligible error rate, while evidence for others (e.g., Aidoc) remains insufficient for comparative assessment [29]. Generally, false positives in such algorithms are associated with intracranial calcifications, while false negatives often arise from chronic occlusions or suboptimal vessel opacification [28,34]. The detection of distal segment occlusions (M2/M3 of the middle cerebral artery and vessels of the posterior circulation, including the PCA) currently represents a key frontier for technological competition. These lesions are inherently more difficult to detect, both for earlier-generation algorithms and less experienced radiologists, primarily due to smaller vessel calibers and greater anatomical variability. Systematic reviews indicate that sensitivity for the M2 segment has historically lagged behind M1, often falling below 80% in older software versions [26]. However, available data suggest rapid progress in this area following successive algorithm updates [26,35].

In the medium vessel occlusion (MVO) landscape, vendors have reported new performance data. In industry-led reports and technical communications from RapidAI (2024/2025), increased detection rates for M2/M3 and posterior fossa occlusions were reported [16,17]. However, because these claims primarily stem from manufacturer-sponsored materials or technical briefs, they should be interpreted with caution pending large-scale, independent validation in peer-reviewed journals [26,35]. Aidoc also maintains a prominent position in the discussion on distal and posterior occlusion detection. While the MVO narrative is often framed as a competition between RapidAI and Viz.ai, retrospective data suggests that for standard LVOs, Aidoc performs comparably to its peers without statistically significant deviation regarding diagnostic accuracy [19]. The literature often highlights Aidoc’s operational profile, particularly regarding its worklist prioritization mechanism. By immediately moving suspected cases to the top of the queue, it facilitates earlier human verification of diagnostically challenging lesions [19]. Similarly, Brainomix is frequently described through an approach that goes beyond simple MVO alerts. Its e-CTA module focuses on detailed vessel visualization and automated marking of suspicious regions, which has translated into high effectiveness in CTA-based detection [32]. Real-world analyses suggest that using Brainomix in tandem with e-ASPECTS enables a more comprehensive evaluation of MVO patients, whose clinical symptoms are often less pronounced than those with LVO [24]. In systems like the NHS, which prioritize integrated assessments of both parenchyma and vasculature alongside workflow efficiency, Brainomix (much like Aidoc) serves as an established alternative to the platforms most visible in vendor communications [33].

### 3.3. Automated Perfusion Assessment (CTP) and Volumetric Discrepancies

Across the included studies, a consistent finding was the lack of full standardization and interchangeability of outputs from automated perfusion (CTP) analysis across different platforms, with direct clinical consequences for patient selection for reperfusion therapy. Within the available literature, the greatest volume of data concerns comparisons between RapidAI and Viz.ai. RapidAI is frequently utilized as a point of comparison in the literature because its algorithms were used for patient selection in landmark clinical trials (DAWN, DEFUSE 3), and the volumetric thresholds applied in practice (e.g., ischemic core volume) are largely calibrated to this system [28]. However, this role is a reflection of its historical presence in trial designs rather than a marker of inherent technological superiority. Furthermore, comparative studies, including those published in peer-reviewed neuroradiology journals, have demonstrated significant volumetric discrepancies between Viz CTP and RapidAI when estimating ischemic core and penumbra volumes in the same patients. This lack of interchangeability highlights the potential for divergent treatment decisions in extreme cases [13,14]. In practical terms, this implies that centers using software other than RapidAI should not automatically apply rigid volumetric thresholds derived from RapidAI-based trials without local validation, as doing so may increase the risk of patient misclassification [13,28].

Against the background of these volumetric disputes, Brainomix is positioned differently, particularly in European literature. Although the platform includes modules supporting vascular and perfusion assessment, the strongest evidence in the reviewed literature relates to an approach based on non-contrast CT (NCCT) and automated e-ASPECTS assessment. Validation studies show high agreement with expert assessment and improved performance compared with less experienced readers in identifying early ischemic changes [10,11]. This “tissue-based” approach may be particularly useful in clinical settings where CTP is not performed routinely for all patients or is affected by technical limitations (e.g., motion artifacts, variable contrast bolus quality), requiring the integration of NCCT and CTA data for therapeutic decisions [12,24,33].

For Aidoc, the literature included in this review indicates a focus on LVO detection sensitivity rather than detailed perfusion volumetry [19]. Regarding perfusion-based methods in general, the literature highlights limitations such as the risk of “ghost core” (false overestimation of ischemic core) due to delayed contrast arrival. Consequently, an integrated approach combining tissue-based measures (e.g., ASPECTS) and CTA-based vascular evaluation is recommended to reduce reliance on a single, potentially unstable volumetric parameter [36].

### 3.4. Non-Contrast CT (NCCT) Assessment: The Role of Brainomix

Regarding non-contrast CT (NCCT) evaluation, Brainomix e-ASPECTS was the most frequently assessed tool, demonstrating the strongest representation in the literature for the automated scoring of early ischemic changes. Multicenter validation studies demonstrate that automated e-ASPECTS achieves high agreement with expert neuroradiologists, reducing subjectivity and inter-rater variability and thereby improving the reproducibility of NCCT interpretation in acute stroke [12]. Available data indicate that Brainomix achieves high diagnostic agreement with expert neuroradiologists, and its generated ‘heatmaps’ serve as valuable decision support aids, particularly in centers with limited neuroradiology expertise where standardized interpretation is critical [10,11,24]. In reports on system-level implementation, especially within the UK NHS, e-ASPECTS is described as an important triage component in settings where perfusion is not routinely performed, enabling rapid selection of patients based on tissue assessment on NCCT complemented by vascular assessment on CTA [33]. RapidAI also offers an automated ASPECTS module, and available studies support its utility in NCCT analysis; however, compared with Brainomix, the RapidAI literature is more heavily focused on perfusion (Rapid CTP) and LVO detection. This indicates that ASPECTS is more often evaluated as part of a broader, integrated protocol (NCCT + CTP) rather than as a stand-alone tool supporting simplified protocols (NCCT + CTA) [28,35]. In contrast, the body of evidence for Viz.ai and Aidoc indicates that these platforms are primarily described through the lens of vascular and organizational solutions, namely rapid detection of LVO and improvements in coordination and case prioritization, while the available literature features a less extensive, dedicated validation corpus for automated, quantitative assessment of ischemic extent on NCCT, comparable to that for Brainomix [7,19,37,38]. Although platforms such as RapidAI or Viz.ai also offer NCCT modules, the evidence base for Brainomix remains notably more extensive in this specific domain, particularly within European healthcare models like the UK NHS [3,24,33,39].

### 3.5. Impact on Workflow and Time Metrics

Workflow-related effects often relate to AI tools improving organization of acute stroke care, including streamlining logistics and reducing key time delays. Although accelerating diagnosis and treatment is a shared objective across platforms, the mechanisms by which this is achieved differ, as reflected in study designs and reported metrics. For Viz.ai, the largest number of studies reported reductions in time to endovascular intervention, particularly as measured by the door-to-puncture (DPT) metric. While independent multicenter data from the VALIDATE study [37] and peer-reviewed clinical trials [40] support these findings, similar (and sometimes broader) clinical savings are also highlighted in manufacturer-sponsored registries and summaries [8,38]. Typically, the implementation of the Viz LVO module has been associated with reductions in treatment times ranging from 11 to 25 min (with selected analyses reporting savings up to 30 min), depending on center characteristics and structure of the stroke pathway [37,38,40]. This effect is attributed to a shift from traditional, sequential communication to a parallel model in which AI-generated alerts and images are delivered directly to the mobile devices of team members (including thrombectomy operators), enabling earlier resource activation even before the formal radiology report is issued [7].

For Brainomix e-Stroke, the literature emphasizes benefits arising primarily from network-level deployment, especially within hub-and-spoke models operating in public healthcare systems. Implementation reports within the UK NHS indicate that a key organizational value lies in standardized assessment (e-ASPECTS/e-CTA), which supports decision-making in regional centers and may reduce suboptimal secondary transfers, improving the use of medical transport and shortening overall ischemic time through faster and more confident patient selection for treatment in referral centers [3,33]. These findings are further supported by real-world evidence analyses, including single-center evaluations reporting diagnostic accuracy [24], as well as broader network-level assessments demonstrating improved stroke pathways and better access to mechanical thrombectomy across integrated delivery networks [33].

From the radiology workflow perspective, relevant evidence comes from direct comparisons between Aidoc and RapidAI. A retrospective comparative analysis showed that Aidoc had shorter processing times, resulting in faster alert generation from the moment images were transferred from the scanner. In addition, the platform is described as a triage tool that integrates with the worklist and automatically prioritizes studies with suspected occlusion by moving them to the top of the reporting queue; this mechanism may be particularly important in high-volume emergency departments with constrained radiology resources [19]. While RapidAI offers comprehensive workflow solutions, in cited comparisons with Aidoc it was sometimes less favorable in terms of raw alert-generation speed, which may be operationally relevant in high-volume environments [19]. At the same time, the literature underscores that RapidAI remains a critical decision-support tool due to its advanced perfusion analysis and established clinical validation, which increase confidence in patient selection in complex scenarios [13,22].

### 3.6. Cost-Effectiveness

Analysis of the available economic evidence indicates that AI systems used in stroke diagnostics, despite high implementation costs associated with subscription-based software-as-a-service (SaaS) models, are generally considered cost-effective, and the potential financial benefits for payers and healthcare systems may substantially exceed licensing costs [27,28]. These conclusions are supported by Health Technology Assessment (HTA) reports, including NICE documents and independent academic analyses, which indicate that the principal source of economic value is not the replacement of radiologist work but rather improved clinical outcomes through the faster and more accurate referral of patients to reperfusion therapies [28,29]. Economic modeling (often based on Markov models) suggests that cost-effectiveness is driven primarily by an increased proportion of patients eligible for mechanical thrombectomy and thrombolysis due to improved detection, as well as by a reduction in long-term disability through shorter treatment delays, translating into better functional outcomes on the modified Rankin Scale (mRS) and lower costs of long-term care and rehabilitation [5,27]. At the system level, this corresponds to favorable incremental cost per quality-adjusted life year (QALY) values, typically within the willingness-to-pay thresholds applied in public healthcare systems [28]. In comparative assessments, the largest body of real-world evidence exists for Brainomix e-Stroke, particularly in the UK NHS. Network-level deployment has been shown to facilitate faster patient identification in regional centres and to facilitate faster reperfusion therapy. Economically, savings are driven primarily by the reduced long-term care for dependent post-stroke patients [3,24,33]. Furthermore, NICE recommendations support its favorable economic profile while advising continued data collection [29]. For Viz.ai, cost-effectiveness is most commonly linked to improved workflow metrics, particularly reductions in door-to-puncture time, which, in economic models, translate into better functional status and lower societal costs, as well as potential optimization of transport logistics and resource utilization [4,8,37]. RapidAI justifies cost-effectiveness primarily through precise patient selection in the late time window based on perfusion analysis, which helps avoid costly futile procedures in patients with extensive infarction while identifying those with substantial penumbra for whom the clinical and economic benefit of thrombectomy is greatest [22]. For Aidoc, despite the absence of dedicated HTA reports in the analyzed literature, available evidence suggests that shorter processing times and worklist prioritization mechanisms may yield operational benefits for hospitals, including increased emergency department throughput and reduced costs associated with diagnostic delays [1,19].

### 3.7. System-Level Limitations and Methodological Heterogeneity

Current studies show that despite the technological maturity of AI tools used in ischemic stroke, their implementation is associated with important system-level limitations and methodological controversies, the profile of which depends on each platform’s functional scope and underlying operating philosophy. The most serious issue described is the lack of full interchangeability of automated perfusion analysis outputs across vendors, particularly in RapidAI versus Viz.ai comparisons. Head-to-head studies suggest that these algorithms rely on different mathematical models for deriving perfusion parameters, resulting in discrepancies in ischemic core and penumbra volume estimates in the same patients; these differences are often statistically significant and do not represent a simple linear shift, making them difficult to predict and adjust for [13,14]. This has direct clinical implications because widely used volumetric thresholds originate from the DAWN and DEFUSE 3 trials conducted using RapidAI software, and uncritical transfer of these thresholds to outputs generated by other platforms may lead to selection errors, including both unjustified exclusion and inappropriate patient selection for treatment with an unfavorable risk-benefit profile [13,28].

Irrespective of vendor, a recurring limitation of automated perfusion analysis is susceptibility to artifacts and the risk of “ghost core,” i.e., false overestimation of the ischemic core. Underlying mechanisms include motion artifacts, hemodynamic disturbances (e.g., low cardiac output), and delayed contrast arrival. These factors can yield perfusion patterns suggestive of irreversible tissue injury, even when the tissue is potentially salvageable [13,28]. Consequently, reliance solely on automated perfusion maps without critical radiologist verification may lead to false-positive diagnoses of extensive infarction and inappropriate therapeutic decisions [28]. In this context, a subset of the literature emphasizes the value of a hybrid approach, whereby CTP results are verified using independent tissue-based measures, and tools strongly oriented toward NCCT, such as Brainomix e-ASPECTS, may serve as a “safety check” when perfusion outputs are uncertain [10,12].

A further set of controversies relates to the quality and independence of evidence, particularly in the detection of medium and more distal vessel occlusions (MVOs). Reports claiming notable improvements in detectability for specific algorithms (e.g., a reported 33% improvement in detectability) largely originate from sponsored studies and manufacturer communications, limiting the ability to draw definitive conclusions regarding generalizability and methodological comparability [16,17]. Independent systematic reviews and validation studies suggest that, although progress in detecting M2/M3 occlusions is evident, real-world clinical benefit may be more variable, and increased sensitivity may be accompanied by a higher false-positive alert burden and the potential for “alert fatigue” in on-call practice [26,35]. In addition, the literature highlights a lack of large, independent prospective studies demonstrating that improved detection of small-vessel occlusions translates into better hard clinical endpoints, such as functional outcome on the mRS, rather than merely increasing diagnostic procedures or escalation of care pathways [21].

Specific limitations also arise from platform-specific functional profiling. For Aidoc, the available studies focus mainly on rapid detection and triage of acute pathology (including LVO) and operational performance metrics, whereas the available literature provides less evidence supporting its utility for advanced, volumetric perfusion-based selection and penumbra assessment comparable to solutions developed by vendors with a strong CTP focus [1,8,19]. For Brainomix, despite a strong evidence base in NCCT assessment and network deployments, a limitation discussed in the literature is the smaller volume of validation data in clinical contexts that rely on strict perfusion criteria and practices typical of certain U.S. centers, potentially limiting direct transferability to care models strongly anchored in RAPID-derived standards [24,33]. Overall, the key limitations include the lack of standardization and interchangeability of CTP outputs across platforms, susceptibility of perfusion analyses to artifacts and “ghost core,” variable evidence quality in the MVO domain, and differences in functional scope across tools. Collectively, these findings support the need for local validation, critical interpretation of outputs, and integrated assessment strategies rather than reliance on a single automatically generated parameter for clinical decision-making [13,16,17,24,25,26,28,33,35]. A comparison of AI systems assisting in acute stroke diagnosis is presented in Table 1.

## 4. Discussion

### 4.1. Clinical Utility: AI as a Diagnostic Safety Net Rather than Labor Substitution

A review of the literature suggests that artificial intelligence systems (Brainomix e-Stroke, Aidoc, RapidAI, and Viz.ai) have moved beyond the experimental phase and have reached a level of technological maturity that has enabled their integration into clinical workflows under clinician oversight. However, it is important to note that these tools do not consistently outperform an experienced neuroradiologist in direct head-to-head comparisons of diagnostic accuracy. Instead, their primary utility appears to lie in triaging suspected cases, supporting workflow prioritization, and providing a diagnostic safety net by standardizing certain aspects of decision-making, particularly in high-volume settings or during on-call hours, when access to subspecialty expertise is often constrained.

It is important to highlight that clinical and technological maturity is not exclusive to the most widely cited platforms (RapidAI, Viz.ai, and Brainomix e-Stroke). Our findings, supported by retrospective comparative analysis, demonstrate that Aidoc performs at a comparable level to widely established platforms, such as RapidAI in LVO detection, with no statistically significant differences in diagnostic accuracy reported in head-to-head comparisons [19]. These findings suggest that Aidoc has reached a stage of development positioning it as a viable alternative for acute ischemic stroke triage. Importantly, while Aidoc demonstrates operational advantages through worklist prioritization, the current literature lacks comparative evidence demonstrating whether this mechanism translates to improved long-term functional outcomes (e.g., mRS) as effectively as the parallel mobile communication model utilized by platforms like Viz.ai [8,19,37].

Clinical data have shown that automated LVO detection systems can achieve clinically relevant sensitivity, effectively functioning as a “digital safety net” that reduces the risk of missed diagnoses due to human fatigue or inexperience [7,8]. This is especially valuable during on-call shifts when immediate neuroradiological expertise may not be available. Observational data from both peer-reviewed studies and real-world implementation reports indicate that AI-supported stroke care coordination platforms may be associated with improvements in selected workflow intervals, such as door-to-referral and team notification intervals [3,37,38]. Nevertheless, it is critical to recognize that no platform is immune to errors; for instance, smaller or distal vessel occlusions may still be missed. Consequently, AI should be regarded not as a final authority, but as a “second pair of eyes” designed to support radiologists [6,43]. In practice, for these tools to be both safe and effective, they must be seamlessly integrated into existing clinical protocols under continuous specialist supervision.

### 4.2. The Problem of Inter-Vendor Variability: Can CTP Results Be Interchanged?

One of the most clinically relevant findings is the lack of standardization in CTP analysis across leading software providers. A 2024 study published in the American Journal of Neuroradiology reported statistically significant differences in volumetric estimates between RapidAI and Viz.ai, even when analyzing the same datasets, highlighting potential variability in clinical interpretation [13,14]. These discrepancies underscore that perfusion parameters, such as ischemic core and penumbra volumes, lack cross-platform interchangeability.

Late-window thrombectomy trials applied automated perfusion imaging and ischemic core estimation to guide eligibility. In the DEFUSE 3 trial, perfusion mismatch criteria with the predefined core volume thresholds were used, whereas the DAWN trial employed a clinical-core mismatch framework stratified by patient age and NIHSS [44]. While these thresholds were historically established using RapidAI software, they should be viewed as methodological benchmarks rather than universal standards applicable to all available platforms [22]. Our review suggests that using alternative algorithms (e.g., Viz.ai or Brainomix) may result in different patient selection outcomes, potentially leading to overestimation or underestimation of core volumes depending on the underlying mathematical model used [13,14].

Consequently, medical centers should not assume interchangeability between these tools without local validation or adjustment of decision-making thresholds [13,14]. We recommend implementing vendor-neutral calibration, shared reporting standards, and uncertainty outputs (e.g., confidence intervals or “low-confidence” flags) to harmonize perfusion results, suggesting a need for consensus guidelines from major stroke societies to establish vendor-neutral standardization. Ultimately, the lack of standardization underscores that no single platform should be regarded as a final diagnostic authority; rather, automated outputs must be critically integrated into clinical context by the treating team to mitigate risk from borderline or discordant estimates.

### 4.3. Expanding to the Distal Vasculature: The MVO Challenge

While the detection of M1 segment LVOs has become routine in numerous stroke pathways, recent development efforts have increasingly focused on distal vessel occlusion (M2/M3 segments, anterior and posterior cerebral arteries). Vendors, notably RapidAI, have promoted their latest algorithms as dedicated solutions for medium vessel occlusion detection in manufacturer communications and company press releases [16,17]. However, independent studies published between 2024 and 2025 suggest that evidence supporting significant performance gains in distal vessels remains limited and methodologically heterogeneous. Although sensitivity for MVO is improving, it remains lower than for LVO detection [26,35]. 

Importantly, the challenge of detecting MVOs may be addressed not only by increasing algorithmic sensitivity, as emphasized in certain vendor-led approaches (RapidAI, Viz.ai), but also through alternative strategies demonstrated in our results, including enhanced vascular visualization (e.g., Brainomix e-CTA) [32] and workflow-based prioritization on the worklist (e.g., Aidoc). Together, these diverse approaches suggest a more pragmatic framework for supporting MVO assessment, shifting the focus from pure automation to workflow-integrated decision support. 

Detection of distal occlusions is associated with an increased risk of error due to anatomical variability and smaller vessel diameters [21]. Ultimately, prospective, independent trials are required to determine whether automated MVO detection translates into improved functional outcomes (mRS), rather than merely increasing diagnostic escalation or intervention rates. Clinical adoption in this frontier should therefore be guided by robust, independently generated evidence rather than marketing-driven performance claims. In this review, we included industry-reported MVO data to map the technological trajectory, balancing these findings against independent validation studies to ensure an objective assessment of clinical readiness. Future studies should consider structuring evaluations around specific tasks (LVO detection, MVO detection, tissue assessment, workflow orchestration), identifying common error modes, and reporting measurable human-AI endpoints such as time-to-human-confirmation, alert burden, and decision-curve analyses to quantify workflow impact and potential alert fatigue.

### 4.4. Standardizing Acute Care in Resource-Limited Settings: The Impact of e-ASPECTS

A notable trend in European literature, particularly from the UK, is the specific role of the Brainomix e-Stroke platform. While American models tend to be focused primarily on perfusion imaging, Brainomix emphasized advanced NCCT analysis using e-ASPECTS algorithms [10,11]. This approach is both more accessible and cost-effective, allowing for rapid patient assessment in smaller centers that may lack advanced perfusion protocols. External validation indicates that e-ASPECTS can support a more consistent assessment of ischemic injury, showing moderate agreement with expert ratings [12,24], and real-world implementation demonstrates its practical utility in routine clinical settings, according to a Health Innovation Oxford report [33]. These findings suggest that Brainomix can play a critical role in supporting standardized stroke assessment, particularly in resource-limited settings, by facilitating timely and reliable clinical decision-making.

### 4.5. Evidence Reliability: The Influence of Industry Sponsorship on Reported Outcomes

A quantitative analysis of the included evidence reveals a significant industry footprint, particularly in the early validation phases of AI platforms. While the majority (86.2%) of the overall literature base remains independent, a discernible pattern emerges in the domain of emerging technologies, such as MVO detection. In this specific area, the most favorable performance claims often originate from manufacturer-sponsored technical briefs or industry-led pilot studies [16,17]. This concentration of industry-led evidence underscores the need for caution when translating these results into clinical protocols without secondary, independent validation [34]. Notably, while manufacturer-sponsored studies often report favorable detectability or performance based on curated datasets, independent head-to-head analyses (e.g., comparing Aidoc and RapidAI) suggest generally comparable performance profiles reported across platforms [19,20]. This reinforces the perspective that workflow-related benefits, such as DPT reductions or worklist prioritization [3,37], are likely more reliable indicators of clinical value than marginal accuracy improvements often touted in industry-led trials. 

Another complicating factor is the rapid pace of software updates; by the time a study is published, the algorithm it evaluated is often superseded by a newer version. This gap between “tested” and “deployed” versions complicates both regulatory oversight and the synthesis of reliable evidence [25]. This underscores why clinicians should remain cautious of data provided by vendors and instead rely on independent Health Technology Assessments (HTA) for implementation decisions [29]. 

Additionally, it is important to recognize the regulatory scope of these tools: according to the U.S. Food and Drug Administration [41], the Viz.ai Contact application was permitted for marketing as a clinical decision support tool designed to analyze CT images and notify specialists of suspected strokes, and it is intended to assist clinicians rather than to replace full clinical evaluation or provide a definitive diagnosis. Ultimately, AI appears to contribute most to stroke care by streamlining clinical workflows and providing a diagnostic safety net, rather than by consistently outperforming human experts in isolated diagnostic tasks.

### 4.6. The Economic Case: Long-Term Savings vs. Immediate Costs

Even with high subscription fees, current economic data suggest that AI in stroke imaging may be cost-effective. Evidence from Health Technology Assessments, systematic reviews, and real-world NHS implementation reports indicates that these technologies could provide both clinical and economic benefits. This perspective is aligned with the NICE DG57 guidance [39], which provides an assessment of AI-driven software modules for stroke decision support (specifically e-ASPECTS and e-CTA), and outlines the evidence limitations and data collection recommendations. Relying on such institutional evaluations helps offset skepticism surrounding industry-sponsored research. By focusing on broader, system-level outcomes, these reports offer a more grounded perspective on whether AI is a viable long-term investment for stroke services. The economic value primarily derives not from replacing clinicians, but from enabling faster patient assessment and treatment, which improves functional outcomes and reduces long-term disability. Consequently, the lifetime care costs for a single patient with severe functional deficits may substantially exceed the annual licensing fees for these platforms, supporting the cost-effectiveness of AI-assisted stroke workflows. However, it must be emphasized that these high SaaS licensing costs represent a disproportionate and significant financial barrier for smaller, non-networked centers with lower patient volumes, where economies of scale cannot be easily achieved [27,28].

### 4.7. Study Limitations and Data Constraints

The scoping review design inherently limits the ability to draw definitive comparative conclusions regarding superiority between platforms. The absence of formal risk-of-bias assessment further restricts interpretability of individual study reliability. Furthermore, the scoping review protocol was not prospectively registered, which should be noted as a limitation regarding methodological transparency. However, this approach allows a broader mapping of emerging evidence in a rapidly evolving technological domain.

Several limitations must be considered when interpreting our findings. The substantial heterogeneity of the included studies, ranging from inconsistent endpoint definitions (e.g., LVO vs. MVO) to varying gold-standard modalities such as CTA and DSA, made a formal meta-analysis unfeasible. This variability complicates any head-to-head comparison of AI platforms. Moreover, we must acknowledge the risk of publication bias, as studies with negative or marginal results are often underrepresented. The high prevalence of industry-sponsored research also raises concerns regarding potential conflicts of interest. To mitigate potential bias, grey literature and industry data were used solely to map emerging technological trends and software updates. In contrast, our conclusions regarding clinical utility and diagnostic accuracy remain anchored in independent, peer-reviewed evidence.

In addition, we identified a lack of head-to-head trials involving all four major AI platforms, making it difficult to rank their performance with confidence. The rapid pace of technological development presents an additional challenge. While this review spans the 2019–2025 period, ongoing algorithmic updates mean that published data may represent a temporary state of the technology, which can already be superseded by newer versions in clinical practice. This latency necessarily affects the interpretation of reported sensitivity and its applicability in real-world settings. 

These limitations highlight the risk of relying solely on retrospective or early-stage data. To advance the field, there is a manifest need for prospective, independently conducted studies with pre-registered endpoints (e.g., DIDO, DTG/DTP, EVT rate, mRS shift, and safety metrics including missed LVO/MVO), rather than solely retrospective validations or vendor-sponsored analyses. Until such evidence is available, the integration of these methods into clinical practice should be approached with caution, ensuring that adoption is guided by robust longitudinal evidence rather than initial performance metrics.

### 4.8. Future Directions: Moving Toward Independent, Multi-Center Trials

Looking ahead, the field should prioritize independent, multicenter trials supported by public funding to minimize potential biases associated with industry sponsorship. In order to bolster causal inference and yield high-quality data, it is imperative to adopt pragmatic and concrete study designs, such as:

1. Prospective “Shadow Mode” Evaluations: Rather than active intervention, AI should initially operate in the background (“silent mode”) to ensure that clinician decision-making remains uninfluenced by the Hawthorne effect or automation bias. By subsequently benchmarking AI outputs against definitive clinical outcomes and expert radiologist interpretations, researchers can more accurately assess both diagnostic performance and potential workflow disruption.
2. Stepped-Wedge Cluster Randomized Rollouts: A sequential implementation across stroke networks allows participating centers to function as their own controls. This design is particularly ethical and practical for stroke networks, as it ensures all centers eventually access the technology while generating robust control data [40].
3. Formal Pre-registration of Endpoints: To mitigate the risk of selective reporting and enable cross-study comparability, it is essential to standardize primary outcomes. This includes time-sensitive metrics such as door-in-door-out (DIDO) and door-to-groin/puncture (DTG/DTP) intervals, alongside EVT utilization rates, functional recovery (mRS), and critical safety indicators, specifically the incidence of missed LVO or MVO cases.

A significant gap remains in our understanding of how these platforms influence long-term functional recovery specifically in MVO populations, a domain largely absent from current literature that necessitates systematic inquiry. Parallel to this, future research should prioritize head-to-head comparisons of different AI-driven alert models, specifically worklist prioritization versus parallel mobile communication, to determine which mechanism most effectively translates to improved long-term functional outcomes (mRS). Establishing vendor-neutral CTP calibration protocols will also be essential to ensure the diagnostic consistency required for robust, multicenter collaboration.

The transition to these more rigorous frameworks will allow the field to move beyond mere workflow efficiency, generating actionable data that directly link AI implementation to measurable improvements in patient safety and clinical outcomes. This evolution is essential if the integration of AI into acute stroke care is to be guided by empirical transparency and clinical utility, rather than a reliance on speculative performance metrics.

## 5. Conclusions

This scoping review demonstrates that current AI platforms function primarily as workflow accelerators and diagnostic safety nets rather than autonomous replacements for radiological expertise. While diagnostic sensitivity for proximal large vessel occlusions (LVO) is reported as comparably high across systems, the detection of distal and medium vessel occlusions (MVO) remains a technological frontier requiring rigorous interpretation and clinician oversight.

A critical finding of this review is the lack of standardization in CT perfusion (CTP) outputs. Volumetric discrepancies between vendors (most notably RapidAI and Viz.ai), indicate that results are not interchangeable, necessitating local validation of treatment thresholds to avoid patient misclassification. Consequently, platform selection is often guided by specific institutional priorities and the prevailing focus of available literature; RapidAI is frequently utilized as a methodological benchmark for perfusion-dependent selection, Viz.ai is commonly associated with reductions in door-to-puncture times through mobile coordination, Brainomix e-Stroke demonstrates unique value in NCCT-based triage and network standardization, while Aidoc is highlighted for its role in worklist prioritization for high-volume settings.

Economically, these systems appear cost-effective, with their value driven primarily by improved access to thrombectomy and long-term functional gains, rather than by staffing reductions. Future implementation must move beyond industry-sponsored validation toward independent, multi-center trials that assess hard clinical endpoints and cross-vendor interoperability.

## Figures and Tables

**Table 1:** Comparison of AI Systems Assisting in Acute Stroke Diagnosis.

Feature/Criterion	RapidAI (iSchemaView) Menlo Park, CA, USA	Viz.ai (Viz.ai, Inc.) San Francisco, CA, USA	Brainomix (e-Stroke) Oxford, UK;	Aidoc Tel Aviv, Israel
**Main Modules & Application**	**Rapid LVO, CTP, ASPECTS, ICH, Angio.** Commonly utilized as a methodological benchmark for perfusion and thrombectomy selection [22].	**Viz LVO, CTP, ICH, Aneurysm.** Primary focus on LVO detection and team coordination (Workflow) rather than just perfusion [37].	**e-ASPECTS, e-CTA, e-CTP.** Emphasized “Tissue-based” approach (NCCT + CTA) supporting decision-making in Hub & Spoke networks [33].	**Stroke (LVO, ICH), PE, C-Spine** [1]. Broad radiological triage (always-on) covering multiple pathologies.
**Occlusion Detection (LVO & MVO)**	**Extensive clinical evidence** for LVO detection. Reported high sensitivity for MVO (per manufacturer technical data [16,17]); independent validation is ongoing [26,35].	**Extensive clinical evidence** for LVO detection (78–97% sensitivity [7,8]). Potential for MVO detection is reported in manufacturer communications and preliminary assessments [18,23], though independent peer-reviewed data for distal segments are more limited [26].	**Evidence includes e-CTA** capabilities for vessel visualization and heatmap marking (MVO) [32].	**Comparable accuracy.** No statistically significant difference in LVO accuracy vs. RapidAI; similar false negative risks [19].
**Perfusion Analysis (CTP) & Evidence Base**	**Established methodological benchmark** in clinical trials (DEFUSE, DEFUSE 3). Treatment thresholds are historically calibrated to this system [22,28].	**Available (Viz CTP).** Significant volumetric discrepancies vs. RapidAI (lack of full interchangeability); risk of divergent eligibility [13,14].	**Available (e-CTP).** Less emphasized in the literature than NCCT; promotes hybrid approach (NCCT + CTA) supported by both vascular and tissue-level validations [10,32].	**Limited.** Insufficient evidence for advanced penumbra/core volumetry in the analyzed literature compared to CTP-focused vendors [1,19].
**Non-Contrast CT Analysis (NCCT/ASPECTS)**	**Available (Rapid ASPECTS).** Often evaluated as part of a broader protocol (CTP + NCCT) rather than a standalone tool [22,35].	**Available**. Less validation data compared to its vascular/organizational modules [7,34].	**Highly documented (e-ASPECTS).** ; acts as a “safety check” when perfusion is uncertain [10,12,33].	**Primary focus on hemorrhage detection (ICH)** with limited emphasis on automated ASPECTS scoring in the current literature [35,41].
**Workflow Impact (Time to Treatment)**	**Demonstrated impact.** Decision support in complex cases, though real-world alert times can vary depending on network stability [22,34,42].	**Documented workflow support** Documented reduction in DPT by 11–25 min via parallel communication and mobile alerts; evidence from multi-center registries [8,37,40].	**Evidence-supported [Network-level].** Standardized assessment in Hub & Spoke models; facilitates faster transfer decisions in NHS settings [24,33,39].	**Strongly documented (Operational).** Efficient worklist prioritization and faster notification processing for LVO compared to traditional radiology workflows [1,19,41].
**Unique Differentiator**	**Clinical trial footprint** (DAWN/DEFUSE 3); extensive CTP calibration [13,22].	**Parallel Workflow & Mobile Coordination:** Shift from sequential to parallel alerts reduces treatment delays [37].	**Broadly documented e-ASPECTS:** Unique value in assessing early ischemic changes on Non-Contrast CT [12,33].	**Platform versatility:** (cross-specialty triage: stroke, PE, fractures) [1].
**Limitations & Controversies**	**Substantial license costs** [22]. A significant proportion of comparative studies are industry-sponsored, which may affect the generalizability of certain performance claims [34].	**Lack of full perfusion interchangeability with RapidAI;** studies report significant volumetric discrepancies in ischemic core/penumbra estimates, potentially affecting patient eligibility [13,14].	**Geographic focus.** Predominance of European/UK-based validation data; fewer studies in U.S. healthcare settings compared to Rapid/Viz [24,33].	**Fewer studies dedicated strictly to stroke pathophysiology** (penumbra/core) and lack of specific HTA reports [1,19,28].
**Mapping the Evidence**	**RCT-level evidence for LVO;** MVO data relies primarily on industry-sponsored studies and lacks large-scale independent prospective trials [15,16,17,26].	**Predominantly Real-World Evidence (RWE),** focus on large-scale workflow and coordination studies [7,37].	**Primarily independent validation** via **NHS/HTA** and major external trials [12,28,33].	**Predominantly retrospective** comparative studies; currently lacks large-scale RCTs [19,20].
**Key References**	[13,16,17,22,26,28,34], [35] *, [42]	[7,8,13,14,18,23,26,34], [37] *, [40]	[10,12], [24] *, [28,32,33,39]	[1,19,20,28], [35] *, [41]
**Evidence Origin & Independence**	**Significant industry involvement.** Historically linked to landmark trials (e.g., DEFUSE 3) [22,35]. A substantial portion of recent MVO data is derived from vendor-initiated studies [16,17].	**Significant industry involvement.** Performance metrics are predominantly reported in manufacturer-sponsored registries and technical briefs focusing on clinical workflow and MVO detection [8,18,37].	**Predominantly independent evidence base.** Evidence base is predominantly characterized by independent NHS-funded studies and Health Technology Assessment (HTA) reports (e.g., NICE) [24,29,33]	**Mixed independence profile.** Primarily evaluated through independent academic retrospective cohorts and studies focusing on multi-pathology triage efficiency [1,19,20].

* Conflict of Interest: Vendor Affiliation.

## Data Availability

No new data were created or analyzed in this study. Platform Access: All reviewed platforms are commercial clinical decision support systems. Publicly available product information and access routes include: Brainomix e-Stroke (https://www.brainomix.com/), RapidAI (https://www.rapidai.com/), Viz.ai (https://www.viz.ai/), and Aidoc (https://www.aidoc.com/). Access typically requires institutional contact and contractual arrangements; these links are provided for transparency and reproducibility.

## References

[1] Heeralal V.T., Chadee S.E., Ilyaev B., Ilyaev R., Ilyayeva S. (2025). Artificial intelligence in stroke care: A narrative review of diagnostic, predictive, and workflow applications. Cureus.

[2] Cimflová P. (2022). Utilization of CTA and CTP in Middle Cerebral Artery Stroke. Ph.D. Thesis.

[3] Nagaratnam K., Neuhaus A., Briggs J.H., Ford G.A., Woodhead Z.V.J., Maharjan D., Harston G. (2023). Artificial intelligence-based decision support software to improve the efficacy of acute stroke pathway in the NHS: An observational study. Front. Neurol..

[4] Liu Z., Leong M.Q., Li N., Teo M.M., Leong W.-L.R., Wong S.C.P., Chew J.S., Saffari S.E., Pang Y.H., Chia G.S. (2024). Reducing Door-to-Puncture Times for Mechanical Thrombectomy in a Large Tertiary Hospital. Neurol. Clin. Pract..

[5] Akay E.M.Z., Hilbert A., Carlisle B.G., Madai V.I., Mutke M.A., Frey D. (2023). Artificial intelligence for clinical decision support in acute ischemic stroke: A systematic review. Stroke.

[6] Vagal A., Saba L. (2022). Artificial Intelligence in “Code Stroke”-A Paradigm Shift: Do Radiologists Need to Change Their Practice?. Radiol. Artif. Intell..

[7] Karamchandani R.R., Helms A.M., Satyanarayana S., Yang H., Clemente J.D., Defilipp G., Strong D., Rhoten J.B., Asimos A.W. (2023). Automated detection of intracranial large vessel occlusions using Viz.ai software: Experience in a large, integrated stroke network. Brain Behav..

[8] Sarhan K., Azzam A.Y., Moawad M.H.E.D., Serag I., Abbas A., Sarhan A.E. (2025). Automated Emergent Large Vessel Occlusion Detection Using Viz.ai Software and Its Impact on Stroke Workflow Metrics and Patient Outcomes in Stroke Centers: A Systematic Review and Meta-analysis. Transl. Stroke Res..

[9] Amukotuwa S.A., Straka M., Dehkharghani S., Bammer R. (2019). Fast automatic detection of large vessel occlusions on CT angiography. Stroke.

[10] Dorochowicz M., Kacała A., Puła M., Korbecki A., Kosikowska A., Tołkacz A., Zimny A., Guziński M. (2025). Assessing the eligibility of Brainomix e-ASPECTS for acute stroke imaging. Front. Neuroinform..

[11] Hoelter P., Muehlen I., Goelitz P., Beuscher V., Schwab S., Doerfler A. (2020). Automated ASPECT scoring in acute ischemic stroke: Comparison of three software tools. Neuroradiology.

[12] Mair G., White P., Bath P.M., Muir K.W., Al-Shahi Salman R., Martin C., Dye D., Chappell F.M., Vacek A., von Kummer R. (2022). External Validation of e-ASPECTS Software for Interpreting Brain CT in Stroke. Ann. Neurol..

[13] Bushnaq S., Hassan A.E., Delora A., Kerro A., Datta A., Ezzeldin R., Ali Z., Anwoju T., Nejad L., Silva R. (2024). A comparison of CT perfusion output of rapidai and viz.ai software in the evaluation of acute ischemic stroke. AJNR Am. J. Neuroradiol..

[14] Alwood B.T., Meyer D.M., Ionita C., Snyder K.V., Santos R., Perrotta L., Crooks R., Van Orden K., Torres D., Poynor B. (2024). Multicenter comparison using two AI stroke CT perfusion software packages for determining thrombectomy eligibility. J. Stroke Cerebrovasc. Dis..

[15] Diagnostic Imaging Comparative AI Study Shows Merits of RapidAI LVO Software in Stroke Detection. https://www.diagnosticimaging.com/view/comparative-ai-study-shows-merits-of-rapidai-lvo-software-in-stroke-detection.

[16] RapidAI Press Release New Study Finds RapidAI Significantly Outperforms Viz.ai in Detecting Medium Vessel Occlusions in Stroke Patients. https://www.rapidai.com/press-release/new-study-finds-rapidai-significantly-outperforms-viz.ai-in-detecting-medium-vessel-occlusions-in-stroke-patients.

[17] RapidAI Press Release RapidAI Reveals Superiority Over Competition in LVO Detection. https://www.rapidai.com/press-release/rapidai-reveals-superiority-over-competition-in-lvo-detection-more-cases-identified.

[18] Viz.ai Neurovascular Impact Continues to Expand. Viz.ai Blog.

[19] Bankston M., Stevens C., Ayub M.A., Strobel J., Arevalo O., Cuellar H., Chokhawala H. (2025). A retrospective analysis comparing AIDoc and RAPIDAI in the detection of large vessel occlusions. Brain Circ..

[20] Park S.H., Han K., Jang H.Y., Park J.E., Lee J.-G., Kim D.W., Choi J. (2023). Methods for clinical evaluation of artificial intelligence algorithms for medical diagnosis. Radiology.

[21] Ospel J.M., Nguyen T.N., Jadhav A.P., Psychogios M.-N., Clarençon F., Yan B., Goyal M. (2024). Endovascular treatment of medium vessel occlusion stroke. Stroke.

[22] RapidAI Review (2024). RapidAI for Stroke Detection: Main Report.

[23] Roustom T. Viz LVO vs. Rapid LVO: Comparing the Accuracy of LVO Detection AIs. Medium. https://medium.com/@tarekroustom_16199/viz-lvo-vs-rapid-lvo-comparing-the-accuracy-of-lvo-detection-ais-e4555b456f4.

[24] Mallon D., Fallon M., Blana E., McNamara C., Menon A., Ip C.L., Garnham J., Yousry T., Cowley P., Simister R. (2024). Real-world evaluation of Brainomix e-Stroke software. Stroke Vasc. Neurol..

[25] Yuba M., Iwasaki K. (2022). Systematic analysis of the test design and performance of AI/ML-based medical devices approved for triage/detection/diagnosis in the USA and Japan. Sci. Rep..

[26] Ghozy S., Azzam A.Y., Kallmes K.M., Matsoukas S., Fifi J.T., Luijten S.P.R., van der Lugt A., Adusumilli G., Heit J.J., Kadirvel R. (2023). The diagnostic performance of artificial intelligence algorithms for identifying M2 segment middle cerebral artery occlusions: A systematic review and meta-analysis. J. Neuroradiol..

[27] van Leeuwen K.G., Meijer F.J.A., Schalekamp S., Rutten M.J.C.M., van Dijk E.J., van Ginneken B., Govers T.M., de Rooij M. (2021). Cost-effectiveness of artificial intelligence aided vessel occlusion detection in acute stroke: An early health technology assessment. Insights Imaging.

[28] Westwood M., Ramaekers B., Grimm S., Armstrong N., Wijnen B., Ahmadu C., de Kock S., Noake C., Joore M. (2024). Software with artificial intelligence-derived algorithms for analysing CT brain scans in people with a suspected acute stroke: A systematic review and cost-effectiveness analysis. Health Technol. Assess..

[29] NICE (National Institute for Health and Care Excellence) Artificial Intelligence (AI) Software to Help Clinical Decision Making in Stroke. https://www.nice.org.uk/consultations/1266/1/committee-discussion.

[30] Tricco A.C., Lillie E., Zarin W., O’Brien K.K., Colquhoun H., Levac D., Moher D., Peters M.D.J., Horsley T., Weeks L. (2018). PRISMA Extension for Scoping Reviews (PRISMA-ScR): Checklist and explanation. Ann. Intern. Med..

[31] Peters M.D.J., Godfrey C.M., Khalil H., McInerney P., Parker D., Soares C.B. (2015). Guidance for conducting systematic scoping reviews. Int. J. Evid. Based Healthc..

[32] Seker F., Pfaff J.A.R., Mokli Y., Berberich A., Namias R., Gerry S., Nagel S., Bendszus M., Herweh C. (2022). Diagnostic accuracy of automated occlusion detection in CT angiography using e-CTA. Int. J. Stroke.

[33] Health Innovation Oxford AI in Health and Care Award—Final Report (e-Stroke Evaluation). https://www.healthinnovationoxford.org/wp-content/uploads/2024/11/e-stroke-evaluation-final-report.pdf.

[34] Wardlaw J.M., Mair G., von Kummer R., Williams M.C., Li W., Storkey A.J., Trucco E., Liebeskind D.S., Farrall A., Bath P.M. (2022). Accuracy of Automated Computer-Aided Diagnosis for Stroke Imaging: A Critical Evaluation of Current Evidence. Stroke.

[35] Yedavalli V., Heit J.J., Dehkharghani S., Haerian H., Mcmenamy J., Honce J., Timpone V.M., Harnain C., Kesselman A., Filly A. (2023). Performance of RAPID noncontrast CT stroke platform in large vessel occlusion and intracranial hemorrhage detection. Front. Neurol..

[36] Busto G., Morotti A., Casetta I., Poggesi A., Gadda D., Ginestroni A., Arcara G., Rustici A., Zini A., Padovani A. (2025). Ct-Perfusion Absolute Ghost Infarct Core Is a Rare Phenomenon Associated with Poor Collateral Status in Acute Ischemic Stroke Patients. J. Clin. Med..

[37] Devlin T., Gao L., Collins O., Heath G.W., Figurelle M., Avila A., Boyd C., Ayub H., Sevilis T. (2024). VALIDATE—Utilization of the Viz.ai mobile stroke care coordination platform to limit delays in LVO stroke diagnosis and endovascular treatment. Front. Stroke.

[38] Viz.ai Synchronizing Stroke Care (Viz LVO Clinical Summary). https://www.viz.ai/wp-content/uploads/2023/05/PRM_0210_Rev_03._Whaley_et_al._Viz_LVO_Clinical_Summary_2020.pdf.

[39] National Institute for Health and Care Excellence (2024). Artificial Intelligence (AI)-Derived Software to Help Clinical Decision Making in Stroke (Guidance DG57).

[40] Martinez-Gutierrez J.C., Kim Y., Salazar-Marioni S., Tariq M.B., Abdelkhaleq R., Niktabe A., Ballekere A.N., Iyyangar A.S., Le M., Azeem H. (2023). Automated large vessel occlusion detection software and thrombectomy treatment times: A cluster randomized clinical trial. JAMA Neurol..

[41] U.S. Food and Drug Administration (2022). FDA Permits Marketing of Clinical Decision Support Software Alerting Providers to Potential Stroke.

[42] Soun J.E., Chow D.S., Nagamine M., Takhtawala R.S., Filippi C.G., Yu W., Chang P.D. (2021). Artificial intelligence and acute stroke imaging. Am. J. Neuroradiol..

[43] Sriwastwa A., Aziz Y.N., Weiss K., Buse R., Zhang B., Demel S.L., Ali A., Voleti S., Wang L.L.-L., Vagal A.S. (2025). Performance of Automated Algorithm in Large and Medium Vessel Occlusion Detection: A Real-World Experience. Am. J. Neuroradiol..

[44] Intercollegiate Stroke Working Party/Royal College of Physicians (2023). National Clinical Guideline for Stroke. https://www.strokeguideline.org/app/uploads/2023/04/National-Clinical-Guideline-for-Stroke-2023.pdf.

